# Structure and dynamics of the course of chronic non-infectious somatic diseases in patients during war events on the territory of Ukraine

**DOI:** 10.1186/s12889-023-16394-0

**Published:** 2023-07-31

**Authors:** Liudmyla Kiro, Maksym Zak, Oleh Chernyshov

**Affiliations:** 1 Department of Therapeutic Disciplines, Institute of Medicine of Petro Mohyla Black Sea National University, Mykolaiv, Ukraine; 2 Department of Pediatric and Surgical Disciplines, Institute of Medicine of Petro Mohyla Black Sea National University, Mykolaiv, Ukraine

**Keywords:** Hypertension, Somatic disorders, Type 2 diabetes, War events

## Abstract

**Background:**

The work studied and compared the dynamics of changes in the course of hypertension, type 2 diabetes, and somatized disorders in Ukrainian population, in the 1st period before war (October 2021 – February 2022) and 2nd period during the war (March -July 2022). The choice of the study of the above-mentioned nosology is due, first of all, to the increase in cases of referrals to outpatient doctors regarding hypertensive crises, the increase in the frequency of patients’ complaints about the lability of glycemic indicators during routine monitoring of glucose levels with ambulatory glucometers, the appearance of chronic pain syndrome of unspecified pathology with the beginning of war events.

**Methods:**

272 people were examined in Mykolaiv Region (Ukraine). The patients were divided into three clinical groups: 1st group − 90 people patients with arterial hypertension; 2nd group – 92 people with type 2 diabetes; the 3rd group – 90 people with somatiform disorders.

**Results:**

In the patients of the 1st group during the 2nd period, amount of people with hypertension of the 1st stage increased in 1.8 times (p = 0.0412), with 2nd stage hypertension increased in 2 times (p = 0.0491) and 3rd stage hypertension increased in 2.5 times (p = 0.0391); somatized disorders in 3rd group also increased in 4.1 times (p = 0.001 < 0.05); in 2nd group recorded an increase in HbA1c by 0.99 ± 0.57 mmol/l (p = 1.795e-07 < 0.05), in comparison with the indicators for the 1st observation period.

**Conclusions:**

The war events in Ukraine not only changed the life of every Ukrainian, but also had a significant impact on the dynamics and structure of chronic somatic diseases, in particular due to an increase in patient referrals for hypertensive crises, exacerbations of type 2 diabetes, somatized disorders, and chronic pain syndrome of unknown etiology. Considering these results, it is important to increase the equipment of regional primary care centers with antihypertensive, hypoglycemic and sedative drugs.

## Background

The events of February 24, 2022, during which the military invasion of the Russian army took place on the territory of Ukraine, forced many of us to experience stress caused by mental trauma, to face depression or post-traumatic stress disorder, to feel the traumatic experience of moving and long-term trips to foreign cities and countries [1–3]. The war also greatly limited the availability of medical care in Ukraine [4–6]. The reason for this, along with the destruction of the medical infrastructure, was the mass departure of primary care doctors and narrow specialists to safe regions, interruptions in the supply of medicines, especially in the first weeks of the war, and the resulting interruptions in treatment, the lack of first-aid kits with the necessary medicines and the formation of stocks of medicines for chronic patients before the start of the war, the closing of private laboratories and medical centers, the impossibility of monitoring the course of the disease and prescribed treatment, the loss of medical documentation, which creates difficulties in obtaining consultations and treatment for displaced persons, uneven distribution of humanitarian (medical) aid to territory of Ukraine [7–10].

Such situations have negative consequences for mental and physical health [11–13]. People with chronic diseases of internal organs have become a particularly vulnerable segment of the population [14–17]. War events affected the increase in the number of visits to outpatient clinics and hospitals for hypertensive crises, recurrence of gastric ulcer disease, exacerbation of chronic pain, lability of glycemic indicators and increase in insulin-resistant forms of type 2 diabetes, etc. Unfortunately, there are not many statistical data on the characteristics of the course and distribution structure of somatic pathology during the war. The work studied and compared the dynamics of changes in the course of hypertension, type 2 diabetes, and somatized disorders in the period before and after February 24, 2022. The choice of the study of the above-mentioned nosology is due, first of all, to the increase in cases of referrals to outpatient doctors regarding hypertensive crises, the increase in the frequency of patients’ complaints about the lability of glycemic indicators during routine monitoring of glucose levels with ambulatory glucometers, the appearance of chronic pain syndrome of unspecified pathology with the beginning of war events.

Given the current military realities, this work is very relevant, because knowledge of the dynamics and structure of the prevalence of diseases makes it possible to rationally redistribute humanitarian and financial aid in the field of medicine, save the costs of local self-government bodies, prevent the development of complications by increasing the supply of necessary medicines, improving equipment and accessibility to receiving qualified medical care in wartime, focusing on the structure of morbidity in the respective territories.

The aim of this study is to analyze and compare the dynamics of changes in the course and structure of chronic somatic diseases before and after the beginning of the military events on the territory of Ukraine.

## Methods

### Dataset, study population

272 patients (136 women and 136 men) were followed up by a family doctor on the basis of the University Clinic of the Petro Mohyla Black Sea National University, in Mykolaive, during both periods of the study, through monthly examinations and examinations of patients. All 272 patients remained in the city during the period of the study, despite the occupation. During the occupation, when access to the university clinic was interrupted, in agreement with the patients, the clinic allocated an ambulance, which included: a family doctor, a laboratory assistant, a nurse, who examined the patient and carried out laboratory tests at home. The schedule of visits was agreed with the management of the clinic and patients. Mobile teams worked in shifts 24 h a day. The age of women ranged from 19 to 60 years (the average age was 41.5 ± 11.2 years), the age of men was from 22 to 60 years (the average age was 42.3 ± 12.3 years). The analysis and comparison of the distribution of the structure of chronic diseases was carried out in two periods: the 1st period, October 2021-February 2022 (before the start of the war in Ukraine); 2nd period: March-July 2022 (during the outbreak of the war in Ukraine). The patients were divided into three clinical groups, depending on the existing concomitant pathology, the information of which was taken from the patient’s outpatient cards: 1st group − 90 people (45 women and 45 men) patients with arterial hypertension (AH) of the I-III stage of the disease, blood pressure (BP), which was from 140/90 mm Hg to 185/105 mm Hg. (average indicator 162/98 mm Hg ± 10/2 mm Hg); 2nd group – 92 people with type 2 diabetes (46 women and 46 men) with fasting capillary blood glucose from 6.20 to 12.01 mmol/l (average fasting glucose 9.15 ± 1.31 mmol/l); The 3rd group – 90 people (45 women and 45 men) with somatiform disorders (this category included patients with anamnestic data on the presence of a somatized disorder, somatiform autonomic dysfunction, and chronic somatiform pain syndrome).

The general clinical examination of the patients consisted of the measurement of anthropometric and physiological indicators, the study of the carbohydrate profile, electrocardiographic examination of the heart (ECG), ultrasound examination of the heart and abdominal organs.

### Definitions

The anthropometric study included: determination of body height and weight, calculation of the body mass index (kg/m^2^), measurement of the circumference of the waist and hips, the index “waist circumference/thigh circumference” (WC/TC); physiological: measurement of blood pressure (presence of arterial hypertension), heart rate, BP.

Assessment of carbohydrate metabolism consisted of: determination of fasting blood glucose with a DIACONT glucometer followed by an oral glucose tolerance test (OGTT) (glucose-1.75 g/kg of body weight, but not more than 75 g), measurement of glycosylated hemoglobin with a DCA Vantage TM analyzer; the study of the level of insulin in the blood with the Eleksys-2010 analyzer, the calculation of the НОMA-IR index was carried out according to the formula: НОМА-IR = fasting insulin*fasting glucose/22.5.

To assess the presence of somatized disorders, the SOMS-2 (Somatized Disorders Questionnaire) adapted to the purpose of the study was used (Screening for Somatoform Symptoms). Patients were asked to answer “yes” or “no” to 53 questions about whether these complaints had occurred in the past 2 years (long or short) or were present. Syndrome-complete somatized disorder is diagnosed > 20 points in men and > 25 points in women.

These studies are characterized by simplicity and the possibility of conducting examination not only in the clinic, but if necessary at home, which is very important given the extreme conditions in which the study was conducted.

### Statistic analysis

Statistical for Windows version 8.0 (License Number: 139-845-755) and computer program “Excel 2010” (Microsoft) were used for statistical data processing. At the first stage of the calculation, descriptive statistics were obtained for indicators measured on a quantitative scale. Such characteristics are: median and average value as measures of position; standard deviation and quartiles as measures of dispersion; minimum and maximum value as indicators of sample size. The Kruskel-Wallis test was used to determine differences between independent groups using nonparametric statistical methods. The obtained results were considered statistically significant at p < 0.05. The relationship between the indicators of the quantitative scale was assessed using the Spearman correlation coefficient (r). The strength of the connection was interpreted as follows: very weak − 0-0.3; weak − 0.3–0.5; medium strength − 0.5–0.7; strong − 0.7–0.9; very strong − 0.9-1.0.

## Results

A comparison of the dynamics of arterial hypertension in the first period (before the start of hostilities) and the second period (March-July 2022) recorded that in patients of the 1st group in the second period (March-July 2022) after the start of the war, the number of patients with hypertension 1st stage increased by 1.8 times (p = 0.0412), referrals of patients with 2nd stage hypertension increased 2 times (p = 0.0491), and 3rd degree hypertension increased in 2.5 times (p = 0.0391). Indicators of optimal pressure < 120/80 mm Hg in patients of the 1st group from March to July 2022 were registered 3.9 times (p = 0.049) less often than in the period from October 2021 to February 2022 (Table 1).

**Table 1:** Blood pressure indicators in patients of the 1st group during the observation period

Blood pressure level (mm Hg) (M ± SEM)	1st group (patients with hypertension), BP average = 162/98 mm Hg ± 10/2 mm Hg, (n = 90)		
	The number of patients with hypertension in the 1st period (October 2021-February 2022), n = 90	The number of patients with hypertension in the 2nd period (March-July 2022), n = 90	The difference between the number of appeals from the AH for the 1st and 2nd periods
	n (%)	n (%)	P
Optimal blood pressure < 120/80	15 (16.6%)	4 (4.2%)	0.049
Normal blood pressure 121–139/81–89	36 (39.6%)	11 (12.5%)	0.0047
1st degree hypertension 140–159/90–99	21 (22.9%)	38 (41.7%)	0.0412
Hypertension of the 2nd degree 160–179/100–109	11 (12.5%)	22 (25%)	0.0491
3rd degree hypertension > 180/110	7 (8.4%)	15 (16.6%)	0.0391

The war events affected also the lability of carbohydrate metabolism indicators obtained during laboratory research. In particular, during the 2nd observation period (March - July 2022) in the patients of the 2nd group, an increase in average fasting glucose was recorded by 0.5 ± 0.72 mmol /l (p = 0.006), an increase in postprandial glucose by 0.85 ± 0.84 mmol/l (p = 7.392e-05 < 0.05); an increase in HbA1c by 0.99 ± 0.57 mmol/l (p = 1.795e-07 < 0.05), compared with the indicators for the 1st observation period (October 2021-February 2022) (Table 2).

**Table 2:** Indicators of carbohydrate metabolism in patients of the 2nd group during the observation period

State indicators of carbohydrate metabolism	2nd group 2 (patients with type 2 diabetes), n = 92		
	Indicators of carbohydrate metabolism for the 2nd observation period (March-July 2022), n = 92	Indicators of carbohydrate metabolism for the 1st period (October 2021-February 2022), n = 92	Differences in carbohydrate metabolism indicators in patients with type 2 diabetes during the 1st and 2nd periods
	M ± SEM	M ± SEM	P
Fasting glycemia, mmol/l	6.13 ± 0.72	5.63 ± 0.57	0.006
Glucose level after 2 h, after consuming 75 g of sugar -containing solution	8.09 ± 1.31	7.24 ± 0.37	7.392e-05 < 0.05
HbA1c, %	6.05 ± 0.73	5.51 ± 0.4	1.795e-07 < 0.05
Insulin, µU/l	15.5 ± 4.74	14.3 ± 2.09	0.1275
HOMA-IR	4.00 ± 1.56	3.57 ± 0.69	0.1471

The war also had a negative impact on the psychoneurological field of patients.

The beginning of the military aggression led to an exacerbation and increase in the symptoms of somatization disorders, mainly due to gastrointestinal and asthenic symptoms. Thus, during the 2nd observation period, patients had stomach and abdominal pain in 2.3 times more often (p = 0.042 < 0.05), flatulence complaints were recorded in 2.2 times more often (p = 0.039 < 0.05), loose stools were observed in 3.4 times more often (p = 0.038 < 0.05), constipation in 3.1 times more often (p = 0.031 < 0.05), palpitations and symptoms of heart failure in 2.3 times more often (p = 0.042 < 0.05), increased fatigue in 2.0 times more often (p = 0.044 < 0.05) than among the same group during the 1st observation period (Table 3).

**Table 3:** The structure of the distribution of somatized disorders (SD) in patients of the 3rd group, depending on the observation period

Major somatized disorders	Leading symptoms in patients with established somatization disorders in the 1st period (October 2021-February 2022), n = 90		Leading symptoms in patients with established somatization disorders in the 2nd period (March-July 2022), n = 90		P-value
	n	%	n	%	P
Absence complaints	58	64.6%	14	15.6%	0.001
Pain in the stomach and abdominal cavity	4	4.4%	9	10.0%	0.042
Flatulence	5	5.6%	11	12.2%	0.039
Loose stool	3	3.3%	10	11.1%	0.038
Fasten	4	4.4%	12	13.3%	0.031
Heart palpitations, interruptions in the region hearts	4	4.4%	9	10.0%	0.042
Pain in hands and feet	3	3.3%	7	7.8%	0.154
Back pain	4	4.4%	8	8.9%	0.190
Increased fatigue	5	5.6%	10	11.1%	0.044

## Discussion

The analysis of the epidemiology of somatic diseases conducted by the Ministry of Health of Ukraine recorded that the number of patients with type 2 diabetes in 2022, compared to 2021 (the first half of the year), increased from 54.5 to 59.5 cases per 100,000 population, the number increased hypertensive crises – from 17.8 to 18.7 and cases per 1,000,000 population. At the moment, 650,000 Ukrainians have turned to psychologists and psychiatrists for help. At the same time, according to the survey data, announced by the Ministry of Health of Ukraine, about 71% of citizens have recently experienced stress or severe nervousness, half of the respondents experience anxiety and tension. Also, according to the Ministry of Health, 20–30% of people who have experienced traumatic events may develop post-traumatic stress disorder (PTSD). In addition, in 5–7 years, the Ministry of Health predicts an increase in the number of drug, alcohol and other addictions. Due to the psycho-emotional stress caused by the war, Ukrainians will age by 10–15 years. “We are not talking about appearance, but diseases that had some kind of age structure will occur 10–15 years earlier than it was before the war,” explained Minister of Health of Ukraine Viktor Lyashko.

The results of the study showed that the stress factors caused by the military events in Ukraine significantly influenced the change in the structure of morbidity in general, mainly due to the increase in outpatient visits for hypertensive crises, type 2 diabetes and somatized complaints. The basis of the pathogenesis of a persistent increase in blood pressure in people during military events is the activation of the sympathoadrenal system, the release of catecholamines (adrenaline, norepinephrine), an increase in the production of corticoliberin and the stimulation of the production of glucocorticoids adrenal glands, which affects the acceleration of heart rate and stimulates vasoconstriction [18–21].

The lability of glycemic indicators in patients with type 2 diabetes mellitus against the background of military events is due to the fact that when we are in a state of shock, proteins of the nervous system begin to enter the circulatory system. Similar substances synthesize substances that produce insulin [22, 23]. The stronger the stress, the more antibodies are produced, which provokes the destruction of cells that produce insulin [24–26]. In addition, a large number of counterinsular hormones, in particular adrenaline, also contribute to hyperglycemia [27–30]. Also, with stress, there is a decrease in the protective functions of the immune system, which makes the patient vulnerable to various diseases [31–34]. An increase in the amount of cortisol affects eating disorders [35–37]. Surveys of our patients have recorded an increase in patients’ cravings for overeating, sweet and fatty, which is due to the development of the emotional type of EB disorder in most patients affected by the war [38, 39]. In our opinion, in addition to the above-mentioned factors, the impossibility of receiving medicines in a timely manner, ignoring monitoring of one’s pressure, and sugar also have an impact.

The interaction and relationship between war and the structure of morbidity is traced in many scientific studies. Thus, Bendavid E, Boerma T, Akseer N, Langer A, Malembaka EB, Okiro EA, estimate that nearly 36 million children and 16 million women were displaced in 2017, on the basis of international databases of refugees and internally displaced populations. Current research provides fragmentary evidence about how armed conflict indirectly increases mortality from somatic diseases and affects the survival chances of women and children through malnutrition, physical injuries, infectious diseases, poor mental health, and poor sexual and reproductive health, but major systematic evidence is sparse, hampering the design and implementation of essential interventions for mitigating the harms of armed conflicts [ Bendavid E, Boerma T, Akseer N, Langer A, Malembaka EB, Okiro EA, Wise PH, Heft-Neal S, Black RE, Bhutta ZA; BRANCH Consortium Steering Committee. The effects of armed conflict on the health of women and children. Lancet. 2021 Feb 6;397(10,273):522–532. doi: 10.1016/S0140-6736(21)00131-8. Epub 2021 Jan 24. PMID: 33,503,456; PMCID: PMC7612212]. The impact of changes in eating behavior, unavailability of insulin is described in detail in the archives of the Public Health Service of Amsterdam. Data were collected from the archives of the Public Health Service of Amsterdam and the diabetes aftercare outpatient clinic (‘Diabetes Nazorg’) in Utrecht, the Netherlands, to determine the incidence of diabetes between 1940 and 1950. The number of outpatient visits for newly- diagnosed diabetes in Amsterdam and Utrecht were used to investigate whether the incidence of diabetes increased during World War II, when food was scarce. The number of outpatient consultations at the Public Health Service of Amsterdam for newly-diagnosed diabetes declined from 3 to 1940 (2% of the total number of consultations) to 140 in 1940 (2%). This figure rose to 112 (21%) in 1949 [Hermanides J, Belqhazi L, Michels RP, Hoekstra JB. [Increasing incidence of type 2 diabetes with lifestyle changes: cues from World War II]. Ned Tijdschr Geneeskd. 2008 Nov 1;152(44):2415-7. Dutch. PMID: 19,055,142].

Many examples from the medical archive (WAR FOOD shortages and diabetes mellitus in Japan. Nutr Rev. 1958 Sep;16(9):273-4. doi: 10.1111/j.1753-4887.1958.tb00800.x. PMID: 13,578,234; DARNALL JR. Hospitalization in European Theater of Operations, U.S. Army, World War II. Mil Surg. 1948 Dec;103(6):426 − 39. PMID: 18,894,575), as our observations indicate the relationship and mutual influence of war on the structure morbidity.

Development somatoform pathologies in our opinion, as well as in the opinion of leading world psychiatrists, conditioned on the basis of a person’s desire to endure a disturbing experience. Interest in studying the dynamics and structure of the course of chronic somatic diseases during hostilities is reflected both in the works of domestic and international researchers [40].

## Conclusions

This is the first epidemiological study that provides an objective assessment of changes in the structure and dynamics of the course of chronic non-infectious disease before and during military events on the territory of Mykolaiv region in Ukraine. The study confirmed that since the beginning of the war, the number of referrals of patients with hypertension of the 1st stage increased by 1.8 times (p = 0.0412), referrals of patients with 2nd stage hypertension increased 2 times (p = 0.0491), and 3rd stage hypertension increased 2.5 times (p = 0.0391). Indicators of optimal pressure < 120/80 mm Hg in patients of the 1st group from March to July 2022 were registered in 3.9 times (D = 0.2205, p = 0.049) less often than in the period from October 2021 to February 2022.

The stress caused by the war also affected the lability of carbohydrate indicators in patients with type 2 diabetes, so the patients of the 2nd group recorded an increase in average fasting glucose values ​​by 0.5 ± 0.72 mmol/l (p = 0.006), an increase in postprandial glucose by 0.85 ± 0.84 mmol/l (p = 7.392e-05 < 0.05); an increase in Hb A1c by 0.99 ± 0.57 mmol/l (p = 1.795e-07 < 0.05), in comparison with the indicators for the 1st observation period (October 2021-February 2022). The beginning of the military aggression led to an exacerbation and increase in the symptoms and heaviness of somatization disorders, mainly due to the predominance of gastrointestinal and asthenic symptoms.

Considering these results, it is important to increase the equipment of regional primary care centers with antihypertensive, hypoglycemic and sedative drugs. In addition, it is important to ensure access and the possibility of obtaining specialized advice from an endocrinologist and a cardiologist.

## Data Availability

All data generated or analysed during this study are included in this published article.

## Abbreviations

AH: Arterial hypertension
BMI: Body mass index
BP: Blood preasure
DM: Diabetes mellitus
FG: Fasting glucose
HbA1c: Glucosylated hemoglobin
HOMA-IR: Homeostatic model assessment for insulin resistance
HR: Heart rate
OGTT: Oral glucose tolerance test
RR: Raspiratory rate
WC/TC: Waist circumference/thigh circumference
WC/TC: Waist circumference/thigh circumference index
